# Construction of the Classification Model Using Key Genes Identified Between Benign and Malignant Thyroid Nodules From Comprehensive Transcriptomic Data

**DOI:** 10.3389/fgene.2021.791349

**Published:** 2022-01-14

**Authors:** Qingxia Yang, Yaguo Gong

**Affiliations:** ^1^ Smart Health Big Data Analysis and Location Services Engineering Lab of Jiangsu Province, Department of Bioinformatics, School of Geographic and Biologic Information, Nanjing University of Posts and Telecommunications, Nanjing, China; ^2^ School of Pharmacy, Macau University of Science and Technology, Macau, China

**Keywords:** classification model, key genes, transcriptomics, combined analysis, thyroid nodules

## Abstract

Thyroid nodules are present in upto 50% of the population worldwide, and thyroid malignancy occurs in only 5–15% of nodules. Until now, fine-needle biopsy with cytologic evaluation remains the diagnostic choice to determine the risk of malignancy, yet it fails to discriminate as benign or malignant in one-third of cases. In order to improve the diagnostic accuracy and reliability, molecular testing based on transcriptomic data has developed rapidly. However, gene signatures of thyroid nodules identified in a plenty of transcriptomic studies are highly inconsistent and extremely difficult to be applied in clinical application. Therefore, it is highly necessary to identify consistent signatures to discriminate benign or malignant thyroid nodules. In this study, five independent transcriptomic studies were combined to discover the gene signature between benign and malignant thyroid nodules. This combined dataset comprises 150 malignant and 93 benign thyroid samples. Then, there were 279 differentially expressed genes (DEGs) discovered by the feature selection method (Student’s *t* test and fold change). And the weighted gene co-expression network analysis (WGCNA) was performed to identify the modules of highly co-expressed genes, and 454 genes in the gray module were discovered as the hub genes. The intersection between DEGs by the feature selection method and hub genes in the WGCNA model was identified as the key genes for thyroid nodules. Finally, four key genes (ST3GAL5, NRCAM, MT1F, and PROS1) participated in the pathogenesis of malignant thyroid nodules were validated using an independent dataset. Moreover, a high-performance classification model for discriminating thyroid nodules was constructed using these key genes. All in all, this study might provide a new insight into the key differentiation of benign and malignant thyroid nodules.

## Introduction

Thyroid nodules are regarded as common clinical problems worldwide, and nearly 50% of the population harbor thyroid nodules (Burman and Wartofsky, 2015; Jasim et al., 2020). For benign thyroid nodules, there is no need to perform any medical treatment if it does not keep growing or cause other problems (Durante et al., 2015). Indeed, less than 10% of patients’ thyroid nodules demonstrate disease progression after a median follow-up of 6 years (Ito et al., 2014). But the thyroid malignancy occurring in only 5–15% of thyroid nodules needed to be treated surgically (Wong et al., 2018). Therefore, to improve treatment efficiency, the main challenge is on how to differentiate the malignant nodules from the majority of benign ones reliably using the diagnostic methods (Cho et al., 2020; Singh Ospina et al., 2020).

Until now, to determine the risk of malignancy, fine-needle aspiration (FNA) with cytologic evaluation remains the diagnostic choice for ≥1.0 cm nodules (Heider et al., 2020). But one-third of thyroid nodules could not be discriminated as benign or malignant correctly (Cibas and Ali, 2009). Over the past decade, molecular testing has developed rapidly to improve the diagnostic accuracy as well as minimize cost and unnecessary testing for indeterminate cases (Roth et al., 2018). Moreover, transcript profiling is a widely used technique to discover the molecular changes. Transcriptomics could obtain information simultaneously based on the abundance of multiple mRNA transcripts for the biological sample (Knyazeva et al., 2020; Moncada et al., 2020). So, the gene signatures based on transcriptomic data could be used to distinguish benign from malignant thyroid nodules efficiently.

Recently, there have been a lot of transcriptomic studies to identify the gene signatures associated with thyroid nodules. For example, Giordano et al. found the three genes (PPARG, AQP7, and ENO3) implicated for the neoplastic mechanism of thyroid follicular carcinomas (Giordano et al., 2006). Wojtas et al. confirmed differential expression of seven genes (CPQ, PLVAP, TFF3, ACVRL1, ZFYVE21, FAM189A2, and CLEC3B) between malignant and benign follicular thyroid tumors (Wojtas et al., 2017). Schulten et al. revealed 55 transcripts (GABBR2, NRCAM, ECM1, HS6ST2, RXRG, *etc*.) differentially expressed between follicular variant of papillary thyroid carcinomas and follicular adenomas of the thyroid (Schulten et al., 2015). Hinsch et al. detected that QPRT was a potential marker for the immunohistochemical screening of follicular thyroid nodules (Hinsch et al., 2009). Although there were various signatures identified in different studies, it was reported that they were difficult to be applied in clinical diagnosis because of the inconsistency and unreliability (Singh Ospina et al., 2020).

The inconsistency among gene signatures from different studies might result from many sources, such as limited number of samples (Schwalbe et al., 2017; Osborn et al., 2018). It is understood that these transcriptomic studies were performed using dozens of samples of thyroid nodules. If the multiple independent studies could be combined as one comprehensive dataset, the sample size could be enlarged and the stability of the gene signatures could be enhanced significantly (Mistry et al., 2013). Moreover, weighted gene co-expression network analysis (WGCNA) could be used to identify the modules of co-expressed genes highly associated with the biological mechanism (He et al., 2019). WGCNA has been widely used to explore biomarkers and therapeutic targets of various diseases (Niemira et al., 2019; Chen et al., 2020). Therefore, it was highly needed to identify key genes between malignant and benign thyroid nodules by WGCNA from a comprehensive dataset.

In this work, five independent transcriptomic studies comprising 150 malignant and 93 benign thyroid nodule samples were combined to discover the gene signatures of thyroid nodules. First, 279 differentially expressed genes (DEGs) were identified by the feature selection method (Student’s *t* test and fold change) after data preprocessing and batch effect removal. And various biological process terms (such as hormone metabolic process, platelet degranulation, and thyroid hormone generation) were enriched using these DEGs. Second, the WGCNA model was constructed to identify significant modules of highly co-expressed genes, and 454 hub genes in the gray module were identified. Third, the intersection between DEGs identified by the feature selection method and the hub genes using the WGCNA model was discovered as the key genes. In order to perform the systematic validation, four key genes participated in the pathogenesis of malignant thyroid nodules were validated by an independent dataset. Finally, a high-performance classification model for discriminating benign and malignant thyroid nodules was constructed using these key genes. All in all, this study might provide a useful classification model for discriminating benign and malignant thyroid nodules.

## Materials and Methods

### Collection of Transcriptomic Data From Multiple Studies

A variety of microarray studies based on thyroid tissue were collected by searching the key word “thyroid nodules” in the Gene Expression Omnibus (GEO) database (Barrett et al., 2013). These collected datasets should meet the following criteria (Yang et al., 2020b): 1) the gene expression profiling was conducted using *cDNA* microarray for “*Homo Sapiens*”; 2) the tissues analyzed were thyroid nodules; 3) raw data could be available for further analysis; and 4) the collected datasets should consist of one group of malignant samples and another group of benign ones. As a result, five independent transcriptomic datasets were collected, and each comprised both benign and malignant thyroid nodules. The detailed information of these five collected datasets is provided in Table 1, including dataset ID, number of samples, microarray platform, and tissue indicated in the original publication and references.

**TABLE 1:** Datasets collected from five independent microarray studies of thyroid nodules (sorted by sample size). Each dataset contained one cohort of malignant and another cohort of another group of benign samples.

Id	No. of samples (malignant: benign)	Platform	Tissue	References
GSE27155	95 (78:17)	HG-U133A	Thyroid tissue	*Clin Cancer Res*
				12 (7): 1983–93, 2006
GSE29315	71 (31:40)	HG-U95Av2	Thyroid tissue	Tomas G, *et al.* unpublished, 2012
GSE82208	52 (27:25)	HG-U133 Plus 2	Thyroid tissue	*Int J Mol Sci*
				18 (6): 1,184, 2017
GSE54958	13 (6:7)	HuGene-1.0 ST	Thyroid tissue	*BMC Genomics*
				16 (S1): S7, 2015
GSE15045	12 (8:4)	ABI Human Genome Survey Microarray v.2	Thyroid tissue	*BMC Cancer*
				9: 93, 2009

### Data Preprocessing and Batch Effect Removal

To enhance the consistency and classification capacity, all datasets in this study (Table 1) were combined to discover the key genes of thyroid nodules. The combination of multiple datasets was carried out in *R* environment (v3.4.3, http://www.r-project.org) (Sepulveda, 2020). The raw data (*CEL* file) of all datasets were read, log-transformed, and normalized using the corresponding *R* package, and all parameters were set as default. All probe sets were then mapped to their corresponding gene names using *Bioconductor* (Tippmann, 2015). The average expression value was retained if one gene was mapped to multiple probes (Yang et al., 2020c). To remove batch effects among five independent datasets, *Z*-score transformation was used to adjust the gene expression levels in each dataset (Yang Q et al., 2019b; Yang et al., 2020a). *Z*-score transformation for each gene could be computed by subtracting the mean of all genes and dividing the difference by the standard deviation of all genes in one experiment. After data transformation, the mean value for each experiment became zero with standard deviation equaling one.

### Differentially Expressed Genes Discovered Between Benign and Malignant Thyroid Nodules

In this study, there were five collected datasets integrated as a comprehensive dataset for discovering signatures. This comprehensive dataset consisted of 150 malignant and 93 benign samples of thyroid nodules. To the best of one’s knowledge, this integrated dataset was the largest transcriptomic dataset in the analysis of thyroid nodules. Based on this comprehensive dataset, the DEGs were discovered using feature selection methods including Student’s *t* test and fold change (FC). For Student’s *t* test, *multtest* package of R language was applied, and the adjusted *p*-value < 0.05 was selected as the cutoff (Yan et al., 2019). The fold change was used to compare the mean expression of each gene between malignant and benign thyroid nodules (Yu et al., 2020). The cutoff level of FC was set to logFC >0.58 (FC > 1.5) or logFC < -0.58 (FC < 0.67). The equation of FC was shown below (as shown in Eq. (1)).

log⁡FC=mean(log⁡2(Malignant Group))−mean(log⁡2(Benign Group)). (Eq.1)

The volcano plot was applied to visualize and demonstrate the DEGs using *ggplot2* package. Then the analysis of gene ontology (GO) enrichment was performed to identify the key biological processes for thyroid nodules (Yang et al., 2019a). Moreover, *GOplot* and *clusterProfiler* packages were used for visualizing the biological processes (BP) of GO enrichment (Yu et al., 2012; Yang et al., 2021). The raw *p*-value < 0.05 of GO terms was considered statistically significant.

### Hub Genes Identified Using Weighted Gene Co-Expression Network Analysis

The *WGCNA* package was applied to establish the scale-free weight gene co-expression networks for thyroid nodules (Langfelder and Horvath, 2008). The unqualified genes were screened out, and the matrix of genes’ similarity by Pearson’s correlation analysis was created. Appropriate soft threshold power (*β*) was applied to strengthen this matrix to a scale-free co-expression network (Yang et al., 2020b). The lowest power was chosen, so the scale-free topology fit index curve flattened out upon reaching a high value. The highly correlated genes were assigned into the same module. As a result, the intersection was obtained between DEGs identified by the feature selection method and hub genes in a key module using the WGCNA model. These genes in the intersection were regarded as the key genes for further validation.

### Validation of the Key Genes Based on the Independent Dataset

A systematic validation was conducted by evaluating the upregulated and downregulated genes based on the independent dataset (GSE34289) (Alexander et al., 2012). This validation dataset consisted of two independent datasets from two different platforms. The first independent dataset was detected based on GPL5175 platform (Affymetrix Human Exon 1.0 ST Array). In this dataset, there were 23 malignant and 26 benign thyroid nodules. The second independent dataset was detected based on GPL14961 platform (Afirma-T Human Custom Array). There were 120 malignant and 198 benign samples in this second independent dataset. In this study, the boxplot was used to demonstrate the differential expression of these key genes between malignant and benign thyroid nodules.

### Construction of the High-Performance Classification Model Using the Key Genes

To construct a classification model for thyroid nodules, four powerful classifiers, namely, support vector machine, linear discriminate analysis, partial least squares, and random forest algorithm, were applied in this study (Orru et al., 2012). The key genes between malignant and benign thyroid nodules were used to discriminate different samples. In the first step, the five-fold cross validation of the comprehensive dataset (Table 1) was performed to validate the performance of this classification model. The accuracy of five-fold cross validation could reflect the quality of the model. In the second step, the comprehensive dataset was set as the training set, and the two independent datasets from GSE34289 were set as the test sets. The performance of the independent test set could accurately reflect the classification ability of the model. This high-performance classification model based on machine learning was constructed for discriminating benign and malignant thyroid nodules.

## Results and Discussion

### Collection of Multiple Transcriptomic Data for Thyroid Nodules

A variety of microarray studies based on thyroid tissue were collected by searching the key word “thyroid nodules” in the GEO database. As a result, five independent transcriptomic studies were obtained, and each comprised a cohort of malignant samples and another cohort of benign samples. The detailed information of these independent datasets is provided in Table 1. Among these studies, the five datasets including 150 malignant and 93 benign thyroid nodules were combined as a comprehensive dataset. The boxplots of five datasets before and after batch effect removal are shown in Supplementary Figure S1. The intensity of all samples before batch effect removal was distributed in the range of 4–15 and fluctuated greatly. After batch effect removal, the intensity of all samples was roughly distributed in the range of -1–1. The stable distribution indicated that the batch effects were well removed in the combined dataset by *Z*-score transformation. After data preprocessing and batch effect removal, the comprehensive dataset with 7,265 genes from five independent studies was applied to discover the key genes of thyroid nodules.

### DEGs of Thyroid Nodules Identified Using the Combined Dataset

**FIGURE 1:**
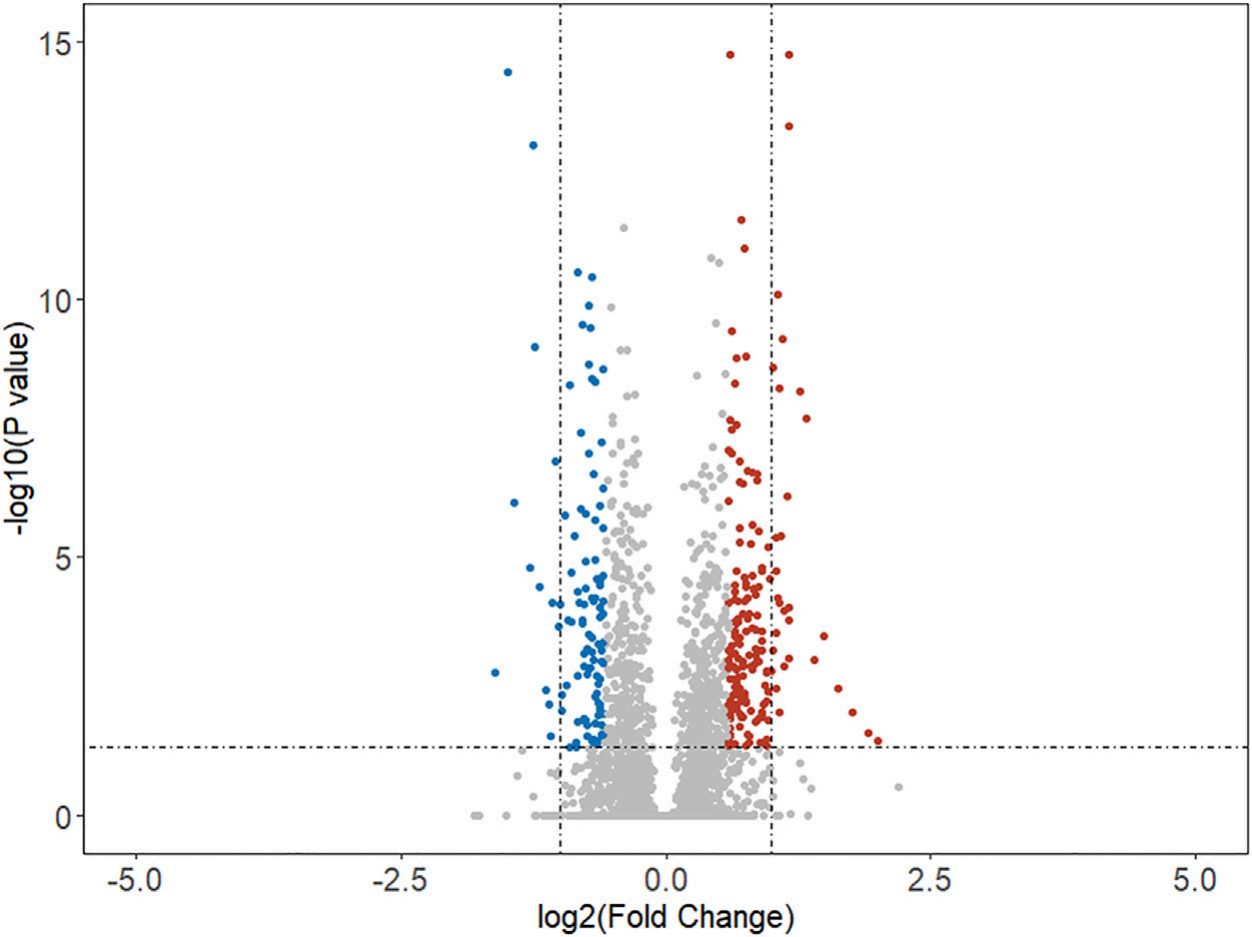
Volcano map of differentially expressed genes in malignant samples compared with benign samples. The horizon line was the cutoff (adjusted *p*-value < 0.05) of Student’s *t* test. The vertical line was the cutoff (logFC >0.58 or logFC < -0.58) of the fold change method. The blue and red dots indicated the downregulated and upregulated genes, respectively.

Based on this comprehensive dataset, the DEGs were discovered using feature selection methods (both Student’s *t* test and fold change). The volcano plot (as shown in Figure 1) illuminated the variation of DEGs in malignant *versus* benign thyroid nodules. The horizon line was the cutoff (adjusted *p*-value < 0.05) of Student’s *t* test. The cutoff levels for the vertical line were set to logFC >0.58 (FC > 1.5) or logFC < -0.58 (FC < 0.67) of fold change. The blue and red dots were used to indicate the upregulated (logFC >0.58) and downregulated (logFC < -0.58) genes, respectively. In this study, 279 DEGs were finally identified by both Student’s *t* test and fold change. The total number of upregulated genes (172 genes) was larger than that of the downregulated ones (107 genes). The top 25 upregulated and downregulated DEGs are shown in Table 2, including the information of entrez ID, gene symbol, adjusted *p*-value, and fold change for each gene. The information of all DEGs is shown in Supplementary Table S1.

**TABLE 2:** Top 25 up- and downregulated DEGs identified by Student’s *t* test and fold change method (logFC >0.58 or logFC < -0.58 and adjusted *p*-value < 0.05) combining all five datasets in Table 1.

ID	Entrez ID	Gene symbol	Adjusted *p*-value	logFC
Table A. The top 25 upregulated genes				
1	9,324	HMGN3	0.035423	1.999879
2	515	ATP5F1	0.02562	1.907751
3	5,800	PTPRO	0.010352	1.767712
4	23576	DDAH1	0.003481	1.626399
5	9,782	MATR3	0.000342	1.498593
6	11167	FSTL1	0.000987	1.408146
7	4,435	CITED1	2.04E-08	1.328755
8	301	ANXA1	5.86E-09	1.273075
9	1803	DPP4	1.81E-15	1.166173
10	55885	LMO3	9.26E-05	1.162304
11	10944	C11orf58	0.00016	1.162246
12	1,001	CDH3	4.16E-14	1.155315
13	722	C4BPA	0.000938	1.154525
14	10178	TENM1	6.51E-07	1.15377
15	439,921	MXRA7	0.001287	1.117048
16	159	ADSS	0.000106	1.113014
17	5,627	PROS1	5.72E-10	1.104001
18	6,447	SCG5	3.80E-06	1.081727
19	7,360	UGP2	7.51E-05	1.076941
20	25797	QPCT	5.05E-09	1.068464
21	1,622	DBI	0.009991	1.065552
22	5,906	RAP1A	6.06E-05	1.055333
23	7,991	TUSC3	7.96E-11	1.05345
24	7,498	XDH	1.86E-05	1.04801
25	10981	RAB32	0.000299	1.046273
Table B. The top 25 downregulated genes				
26	4,703	NEB	3.90E-06	−0.8582
27	432	ASGR1	2.01E-05	−0.89599
28	1805	DPT	0.00018	−0.8994
29	4,494	MT1F	4.58E-09	−0.91087
30	219,333	USP12	0.047108	−0.9167
31	2,117	ETV3	0.000167	-0.93059
32	6,722	SRF	0.003049	−0.94275
33	1,381	CRABP1	1.48E-06	−0.95542
34	6,921	TCEB1	0.004592	-0.98698
35	2,323	FLT3LG	0.009582	−0.98782
36	1,299	COL9A3	8.03E-05	-1.00485
37	4,713	NDUFB7	0.000215	−1.00738
38	4,495	MT1G	1.39E-07	-1.05177
39	9,265	CYTH3	7.71E-05	−1.07064
40	8,458	TTF2	0.030282	−1.09564
41	968	CD68	0.007163	−1.11098
42	6,624	FSCN1	0.003741	−1.12761
43	4,920	ROR2	3.74E-05	−1.19808
44	2,167	FABP4	8.24E-10	−1.24181
45	744	MPPED2	1.02E-13	−1.25312
46	3,292	HSD17B1	1.63E-05	-1.28357
47	1,014	CDH16	3.65E-16	−1.33575
48	1,733	DIO1	8.64E-07	−1.42927
49	7,173	TPO	3.90E-15	−1.49917
50	9,351	SLC9A3R2	0.00174	−1.61953

### GO Enrichment Analysis Using DEGs of Thyroid Nodules

GO enrichment analysis is ubiquitously used for interpreting high throughput molecular data and underlying biological phenomena of experiments (Tomczak et al., 2018). For a set of genes, an enrichment analysis will find which GO terms are overrepresented using annotations for the gene set. GO enrichment analysis for the DEGs was performed in this study. Using the DEGs between malignant and benign thyroid nodules, the enrichment analysis included the BP (biological process), MF (molecular function), and CC (cell component) terms. The detailed information of GO ID, description, *p*-value, name, and the number of genes is shown in Supplementary Table S2.

**FIGURE 2:**
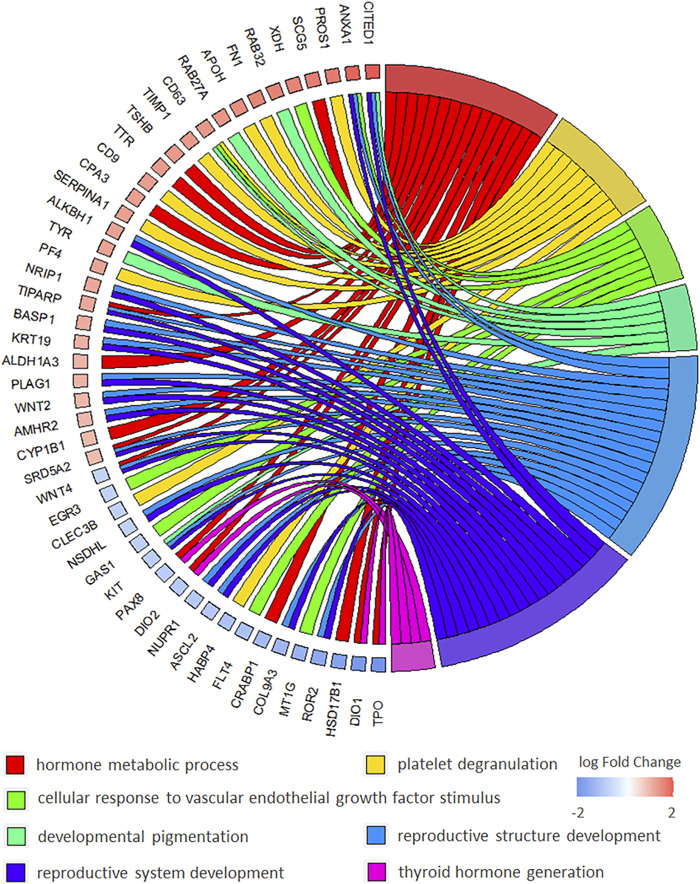
Chord diagram of BP (biological process) of GO enrichment to explain the relationship between BP terms and DEGs in malignant *versus* benign thyroid nodules.

Particularly, multiple biological processes were enriched to interpret the biological mechanism of malignant thyroid nodules. The chord diagram of BP enrichment (as interpreted in Figure 2) was applied to explain the relationship between DEGs and BP terms. It was reported that these BP terms were associated with the biological mechanism of thyroid nodules. For example, there were 15 DEGs enriched in the hormone metabolic process, and the association with thyroid cancer has been reported (Han et al., 2018). The platelet degranulation enriched by 10 DEGs was discovered in papillary thyroid carcinoma using the biomarkers (Wu et al., 2018). The concentration of the vascular endothelial growth factor was increased and stimulated endothelial cell proliferation in the cyst fluid of enlarging and recurrent thyroid nodules (Sato et al., 1997). It was reported that patients with spotty skin pigmentation had a predisposition toward the development of thyroid abnormalities (Courcoutsakis et al., 2009). It was found that low thyroid hormones might have implications for reproductive health, so the reproductive structure development and reproductive system development might be affected in thyroid nodules (Medda et al., 2017). The thyroid hormone generation reported that the significant biologic process was involved in thyroid cancers (Durante et al., 2018).

### Construction of the WGCNA Network and Identification of the Gene Co-Expression Module

**FIGURE 3:**
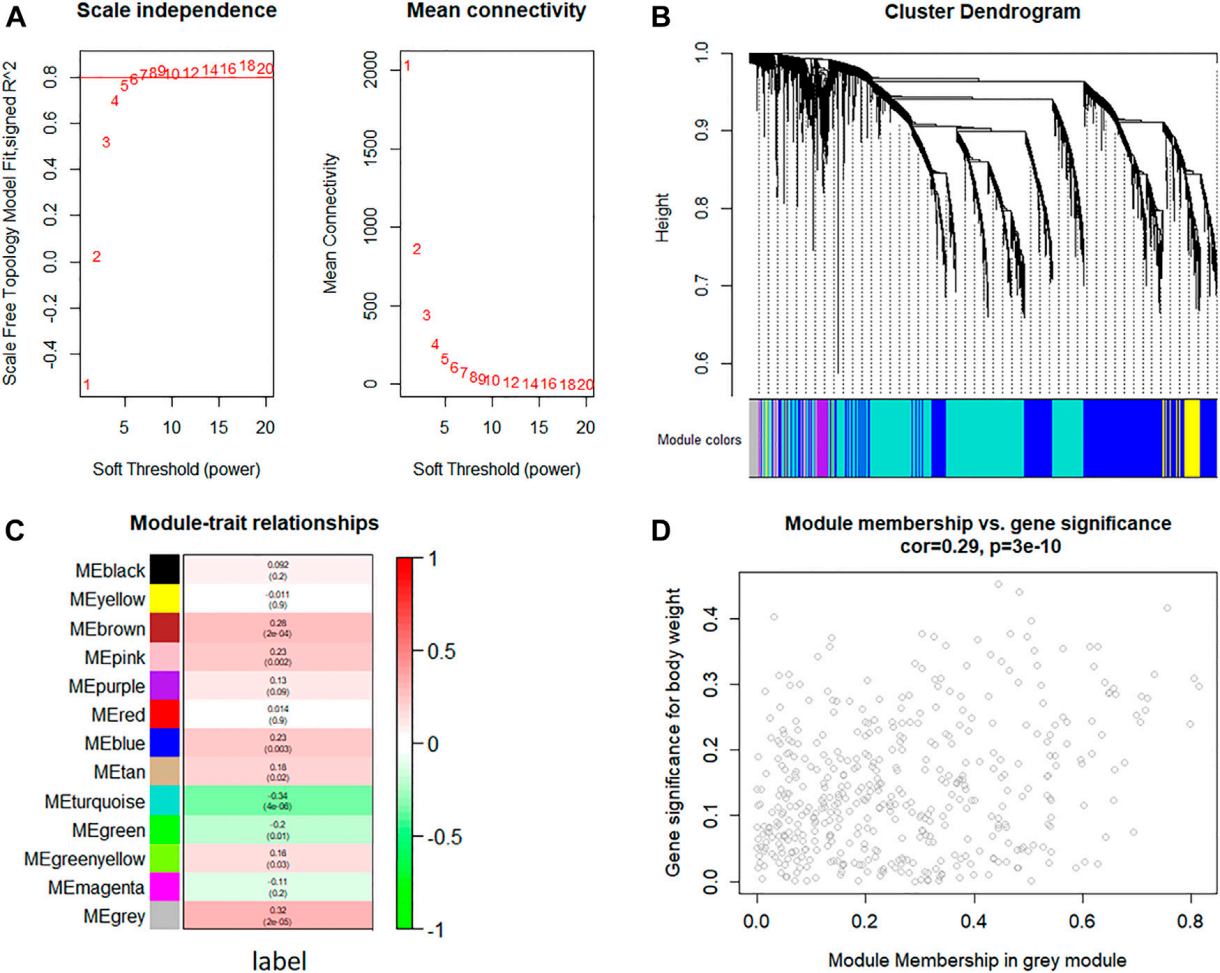
Weighted gene co-expression network analysis of gene expression between malignant and benign thyroid nodules. **(A)** Analysis of the scale-free topology fit index and the mean connectivity for various soft threshold powers (*β*) for the genes, **(B)** dendrogram of all expressed genes clustered based on a dissimilarity measure, **(C)** heatmap of module–trait relationships depicting correlations between module eigengenes and phenotypic traits (the label of malignant and benign thyroid nodules). Numbers correspond to the correlation and the *p*-value in parentheses. The degree of correlation is illustrated with the color legend, and **(D)** identification of hub genes using the scatterplot of module eigengenes in the gray co-expression module.

The WGCNA network was constructed to identify the gene co-expression module (as shown in Figure 3). The value of power (10) was selected as the soft-threshold power to ensure scale-free (*R* ^*2*^ = 0.8) networks using the *WGCNA* package (Figure 3A) because it reached the plateau at power 10 from the scale-free topology plot and mean connectivity plot. Genes with similar expression patterns were clustered into co-expression modules. Different modules were shown in different colors, and 13 modules were identified totally (Figure 3B). The heatmap of module–trait relationships was applied for depicting correlations between module eigengenes and phenotypic traits (the label of malignant and benign thyroid nodules). As shown in Figure 3C, the numbers correspond to the correlation, and the *p*-values were set in parentheses. Moreover, the degree of correlation was illustrated with the color legend. Here, the gray module was the most correlated one with malignant thyroid nodules (*R* = 0.32, *p*-value = 2 × 10^–5^). Hence, the gray module was used for the identification of the hub genes. Hub genes in the co-expression network were characterized by high intra-modular connectivity measured by the value of gene significance and module membership. The scatterplot of module eigengenes related to malignant thyroid nodules in the gray co-expression module (*R* = 0.29, *p*-value = 3 × 10^–10^) is shown in Figure 3D. As a result, 454 genes in the gray module highly correlated with gene significance were identified as hub genes using WGCNA.

### Validation of the Key Genes Using the Independent Datasets

**FIGURE 4:**
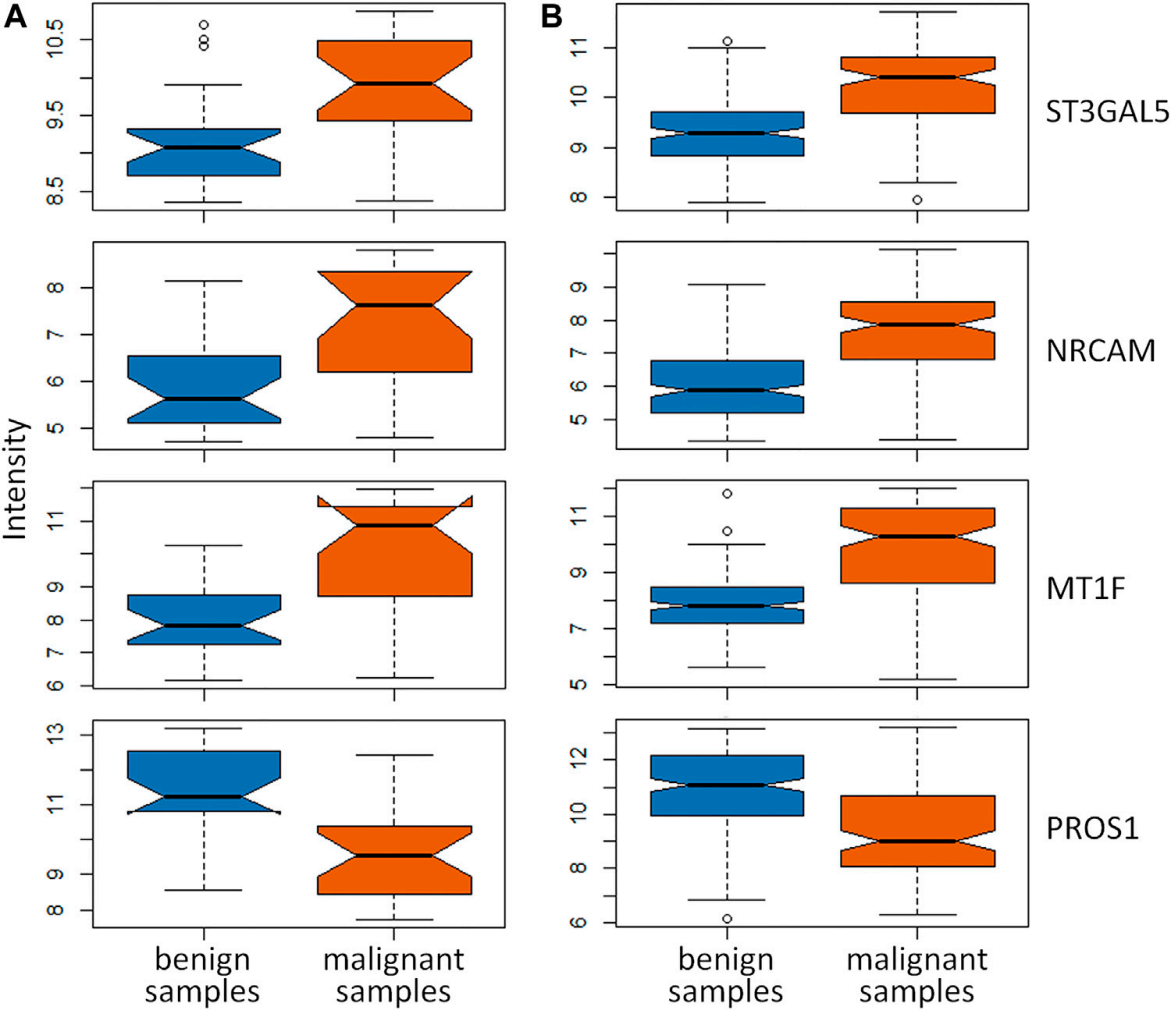
Validation of key genes identified by both DEGs identified by the feature selection method and hub genes in the gray module using WGCNA. The boxplots of these key genes between malignant and benign thyroid nodules were validated in the independent dataset detected by **(A)** GPL5175 and **(B)** GPL14961 platforms.

In this study, there were 19 overlapping genes in the intersection between 279 DEGs identified by the feature selection method and 454 hub genes in the gray module totally. To validate these overlapping genes, two independent datasets from GSE34289 were applied to perform the systematic validation (Alexander et al., 2012). In this validation dataset, there were 23 malignant with 26 benign samples and 120 malignant with 198 benign samples form GPL5175 and GPL14961 platforms, respectively. The boxplots (as shown in Figure 4) were used to demonstrate the key genes between malignant and benign thyroid nodules. Among the 19 overlapping genes, there were four key genes expressed in the independent dataset, and the dysregulation of these key genes was validated. As shown in Figure 4, the significant differences of three upregulated genes (ST3GAL5, NRCAM, and MT1F) and one downregulated gene (PROS1) were indicated in these boxplots obviously for the independent data detected from GPL5175 (Figure 4A) and GPL14961 platforms (Figure 4B), respectively.

As a result, these four key genes were effectively validated as the important ones participated in the pathogenesis of thyroid nodules. It was reported that the specific genetic variants of ST3GAL5 in patients with thyroid-associated ophthalmopathy were discovered (Park et al., 2017). Górka et al. provided the first evidence that NRCAM is overexpressed in papillary thyroid carcinomas, and the upregulation of NRCAM was implicated in the pathogenesis and behavior of papillary thyroid cancers (Gorka et al., 2007). It was reported that MT1F might contribute to thyroid carcinogenesis and potentially serve as a diagnostic marker in distinguishing benign from malignant lesions (Kim et al., 2010; Wojtczak et al., 2017). In the previous studies, PROS1 was reported as the biomarker significantly related to thyroid nodules’ malignancy (Griffith et al., 2006; Wu et al., 2020). In this study, these four key genes (ST3GAL5, NRCAM, MT1F, and PROS1) were discovered for distinguishing malignant from benign thyroid nodules.

### Construction of the High-Performance Classification Model Using the Key Genes

**FIGURE 5:**
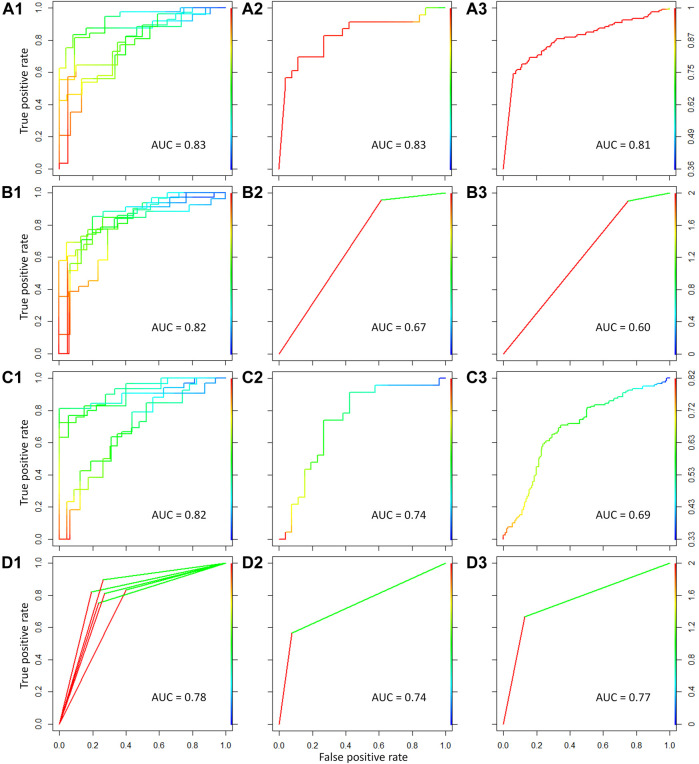
Classification model constructed for discriminating malignant from benign thyroid nodules using four different machine learning methods. The four methods referred to support vector machine, linear discriminate analysis, partial least squares, and random forest algorithm from top to bottom. The ROC curves and AUC values for the five-fold cross validation were shown in **(A1–D1)** for the comprehensive dataset using the four methods. The ROC curves and AUC values for the first independent test set were shown in **(A2–D2)**. The ROC curves and AUC values for the second independent test set were shown in **(A3–D3)**.

To distinguish malignant from benign thyroid nodules, four popular machine learning methods were applied to construct the classification model in this study. These methods included support vector machine, linear discriminate analysis, partial least squares, and random forest algorithm. The key genes between benign and malignant thyroid nodules were used to discriminate different samples. For the comprehensive dataset in Table 1, the five-fold cross validation was first performed to validate the performance of this classification model. As shown in Figure 5A1, 5B1, 5C1, and 5D1, the values of area under the ROC curve (AUC) were 0.83, 0.82, 0.82, and 0.78 for the five-fold cross validation using four different machine learning methods, respectively. Moreover, the high performance of the independent test sets could accurately reflect the ability of the classification model. The comprehensive dataset was set as the training set, and the test sets consisted of two parts detected by GPL5175 and GPL14961 platforms from the independent dataset (GSE34289). As displayed in Figure 5A2, 5B2, 5C2, and 5D2, the AUC values of the ROC curve for the first independent test set were 0.83, 0.67, 0.74, and 0.74 by four machine learning methods, respectively. As shown in Figure 5A3, 5B3, 5C3, and 5D3, the AUC values for the second independent test set were 0.81, 0.60, 0.69, and 0.77 by four machine learning methods, respectively.

As shown in Figure 5, for the five-fold cross validation, the performances (AUC >0.8) of the classification model were outstanding using support vector machine, linear discriminate analysis, and partial least squares. However, the classification models of support vector machine and random forest (AUC >0.7) have shown more excellent performances than the other methods for the two independent test sets. Therefore, the high-performance classification model using support vector machine was recommended for discriminating malignant from benign thyroid nodules based on both five-fold cross validation and independent test.

Until now, it fails to discriminate as benign or malignant in one-third of thyroid nodules using FNA with cytologic evaluation. To save medical costs and improve the diagnostic accuracy, the high-performance classification model constructed in this study could be applied before FNA. For the thyroid nodule patients, the expression of four key genes could be detected. Then, this sample could be classified as benign or malignant thyroid nodules based on the classification model. If the patient was classified as a malignant thyroid sample, it was highly necessary to make a definite diagnosis using FNA with cytologic evaluation. If the patient was classified as a benign sample based on the classification model, the necessity of the FNA could be determined depending on the specific conditions. In the future, selection method, the high-performance classification model is expected to be applied for clinical diagnosis and management for malignant and benign thyroid nodules.

## Conclusion

In this study, a comprehensive dataset including 150 malignant and 93 benign samples was collected to discover the gene signature of thyroid nodules. Then, 279 DEGs were identified by the feature selection method (Student’s *t* test and fold change). Then, the WGCNA network was performed to identify modules of highly co-expressed genes, and 454 genes were discovered as the hub genes. As a result, the intersection between the DEGs and the hub genes was identified as the key genes. Using the independent dataset, three upregulated genes (ST3GAL5, NRCAM, and MT1F) and one downregulated gene (PROS1) were effectively validated. Moreover, the high-performance classification model was constructed for discriminating malignant from benign thyroid nodules. However, certain limitations still exist in this study. The number of samples for identifying and validating key genes was still needed to be increased. In the future, the key genes and classification model could be further verified based on the experimental data.

## Data Availability

The original contributions presented in the study are included in the article/Supplementary Material; further inquiries can be directed to the corresponding author.
