# Supplementary material for: Construction of the Classification Model Using Key Genes Identified Between Benign and Malignant Thyroid Nodules From Comprehensive Transcriptomic Data
Source: Front Genet. 2022 Jan 14;12:791349. doi: 10.3389/fgene.2021.791349 (PMC8795894; doi:10.3389/fgene.2021.791349)
Supplement: Supplementary file 1 [file DataSheet1.pdf]

Supplementary Information

Construction of the Classification Model Using Key Genes Identified between Benign and Malignant Thyroid Nodules from Comprehensive Transcriptomic Data

Qingxia Yang[1], Yaguo Gong[2,*]

[1] Department of Bioinformatics, Smart Health Big Data Analysis and Location Services Engineering Lab of Jiangsu Province, School of Geographic and Biologic Information, Nanjing University of Posts and Telecommunications, Nanjing, 210023, China.

[2] School of Pharmacy, Macau University of Science and Technology, Macau, China.

* Corresponding author: Dr. Yaguo Gong (gongyglab@gmail.com).

Running Title: Classification Model for Thyroid Nodules

Supplementary Table S1. The 279 DEGs (differentially expressed genes) identified by student's *t* test and fold change method (logFC >0.58 or logFC <-0.58 and adjusted *p*-value <0.05) from the combined dataset in **Table 1**.

ID	Entrez ID	Gene Symbol	Adjusted *p*-value	logFC
1	9324	HMGN3	0.035423	1.999879
2	515	ATP5F1	0.02562	1.907751
3	5800	PTPRO	0.010352	1.767712
4	23576	DDAH1	0.003481	1.626399
5	9782	MATR3	0.000342	1.498593
6	11167	FSTL1	0.000987	1.408146
7	4435	CITED1	2.04E-08	1.328755
8	301	ANXA1	5.86E-09	1.273075
9	1803	DPP4	1.81E-15	1.166173
10	55885	LMO3	9.26E-05	1.162304
11	10944	C11orf58	0.00016	1.162246
12	1001	CDH3	4.16E-14	1.155315
13	722	C4BPA	0.000938	1.154525
14	10178	TENM1	6.51E-07	1.153477
15	439921	MXRA7	0.001287	1.117048
16	159	ADSS	0.000106	1.113014
17	5627	PROS1	5.72E-10	1.104001
18	6447	SCG5	3.80E-06	1.081727
19	7360	UGP2	7.51E-05	1.076941
20	25797	QPCT	5.05E-09	1.068464
21	1622	DBI	0.009991	1.065552
22	5906	RAP1A	6.06E-05	1.055333
23	7991	TUSC3	7.96E-11	1.05345
24	7498	XDH	1.86E-05	1.04801
25	10981	RAB32	0.000299	1.046273
26	4712	NDUFB6	0.003468	1.036294
27	2335	FN1	4.05E-06	1.035339
28	10289	EIF1B	0.000625	1.014718
29	4753	NELL2	2.00E-09	1.003549
30	10190	TXNDC9	0.001618	0.995109
31	7756	ZNF207	2.64E-05	0.976303
32	10551	AGR2	6.43E-06	0.96909
33	732	C8B	0.00415	0.963064
34	350	APOH	0.014718	0.96114
35	892	CCNC	0.001779	0.955944
36	3824	KLRD1	0.035056	0.95538

37	5378	PMS1	0.001573	0.952578
38	4184	SMCP	0.006308	0.950243
39	9512	PMPCB	0.043916	0.944831
40	5423	POLB	0.002997	0.938214
41	11171	STRAP	0.007299	0.919299
42	1994	ELAVL1	0.01265	0.913464
43	5651	TMPRSS15	0.010121	0.911664
44	10346	TRIM22	0.000618	0.907274
45	90338	ZNF160	0.000406	0.906331
46	7037	TFRC	0.000276	0.90369
47	5873	RAB27A	1.84E-05	0.901541
48	11113	CIT	1.64E-05	0.898826
49	11321	GPN1	0.039795	0.887767
50	967	CD63	0.000659	0.879819
51	5597	MAPK6	3.13E-06	0.876208
52	7076	TIMP1	3.70E-05	0.87375
53	7252	TSHB	0.001033	0.87295
54	51101	ZC2HC1A	0.0133	0.870748
55	55023	PHIP	2.34E-07	0.865691
56	2059	EPS8	3.23E-07	0.862314
57	9414	TJP2	0.00106	0.857954
58	7276	TTR	0.000134	0.85592
59	2267	FGL1	0.015835	0.851886
60	8481	OFD1	0.001143	0.846666
61	1278	COL1A2	0.000588	0.842995
62	928	CD9	0.000262	0.841572
63	55251	PCMTD2	0.000778	0.838758
64	7818	DAP3	5.35E-05	0.838632
65	9168	TMSB10	4.16E-05	0.826025
66	765	CA6	3.89E-05	0.825968
67	23095	KIF1B	0.000241	0.820109
68	1359	CPA3	2.28E-06	0.818828
69	5265	SERPINA1	2.31E-05	0.817422
70	23362	PSD3	2.28E-07	0.811706
71	8846	ALKBH1	0.001502	0.811226
72	960	CD44	0.009579	0.803231
73	3759	KCNJ2	5.67E-06	0.800751
74	7299	TYR	0.001273	0.799669
75	5858	PZP	0.02972	0.786161
76	57035	RSRP1	0.000778	0.784518
77	6507	SLC1A3	0.000127	0.782141

78	5196	PF4	0.038719	0.780987
79	6947	TCN1	2.13E-07	0.776701
80	910	CD1B	6.19E-05	0.774637
81	51066	SSUH2	0.000158	0.773262
82	9218	VAPA	0.027383	0.767835
83	1848	DUSP6	3.32E-05	0.762232
84	6271	S100A1	0.044205	0.758486
85	10577	NPC2	1.23E-09	0.756935
86	7101	NR2E1	0.000264	0.752901
87	8204	NRIP1	3.71E-05	0.752757
88	23180	RFTN1	0.006735	0.751887
89	10099	TSPAN3	0.006228	0.748554
90	2524	FUT2	0.004373	0.74748
91	10050	SLC17A4	6.96E-05	0.746404
92	8989	TRPA1	0.005963	0.741472
93	6695	SPOCK1	2.44E-05	0.739565
94	1044	CDX1	0.004792	0.738207
95	25976	TIPARP	1.03E-11	0.737825
96	10205	MPZL2	0.012777	0.733413
97	1829	DSG2	0.001937	0.73068
98	5016	OVGP1	0.001261	0.727112
99	10409	BASP1	3.57E-07	0.72472
100	8754	ADAM9	0.000129	0.723038
101	5273	SERPINB10	0.001077	0.721322
102	6385	SDC4	2.79E-12	0.718138
103	9077	DIRAS3	0.012556	0.713309
104	689	BTF3	0.005993	0.710015
105	6281	S100A10	1.35E-07	0.702519
106	26659	OR7A5	0.011039	0.700997
107	1809	DPYSL3	0.000475	0.700883
108	2842	GPR19	3.36E-07	0.6965
109	1068	CETN1	0.003572	0.695286
110	214	ALCAM	0.018923	0.694906
111	3489	IGFBP6	2.75E-06	0.691788
112	1315	COPB1	5.26E-06	0.690871
113	5431	POLR2B	0.000372	0.690003
114	9648	GCC2	0.000155	0.688346
115	3426	CFI	0.000175	0.685456
116	11122	PTPRT	0.000422	0.681387
117	22948	CCT5	0.002134	0.676653
118	9969	MED13	7.31E-05	0.676161

119	8714	ABCC3	1.78E-16	0.67522
120	6005	RHAG	0.000897	0.675176
121	3880	KRT19	2.60E-08	0.673278
122	10924	SMPDL3A	0.004666	0.671237
123	23397	NCAPH	1.85E-05	0.670533
124	8444	DYRK3	0.008629	0.66916
125	26716	OR2H1	0.00278	0.666886
126	26046	LTN1	0.006889	0.666612
127	8288	EPX	0.001537	0.662731
128	10930	APOBEC2	0.000197	0.662694
129	23231	SEL1L3	1.34E-09	0.65943
130	4152	MBD1	0.000759	0.656896
131	959	CD40LG	0.041309	0.656646
132	1272	CNTN1	0.003268	0.655128
133	1781	DYNC1I2	0.000344	0.653878
134	220	ALDH1A3	6.50E-05	0.653545
135	9550	ATP6V1G1	3.59E-05	0.652118
136	8542	APOL1	4.53E-05	0.647395
137	10092	ARPC5	0.000267	0.646439
138	5324	PLAG1	4.17E-09	0.644433
139	22876	INPP5F	0.000184	0.644166
140	1267	CNP	0.00462	0.641788
141	5411	PNN	0.008101	0.633431
142	8747	ADAM21	0.002226	0.630448
143	7472	WNT2	0.001328	0.630211
144	9793	CKAP5	0.007447	0.627886
145	6554	SLC10A1	0.000755	0.623376
146	10782	ZNF274	9.59E-08	0.622719
147	5791	PTPRE	4.13E-10	0.6212
148	8869	ST3GAL5	3.23E-08	0.620601
149	9061	PAPSS1	0.000521	0.616568
150	5593	PRKG2	0.008555	0.614311
151	269	AMHR2	0.041574	0.613742
152	671	BPI	0.030166	0.613295
153	6906	SERPINA7	0.01191	0.611129
154	4076	CAPRIN1	0.005785	0.609744
155	9407	TMPRSS11D	0.020674	0.606399
156	64285	RHBDF1	0.002236	0.604996
157	3698	ITIH2	0.002248	0.604219
158	1545	CYP1B1	2.18E-08	0.602743
159	10200	MPHOSPH6	0.013756	0.60128

160	7039	TGFA	1.71E-15	0.601049
161	2554	GABRA1	0.000197	0.600628
162	4077	NBR1	0.027874	0.600467
163	6582	SLC22A2	0.04372	0.597038
164	2597	GAPDH	0.007192	0.596042
165	347902	AMIGO2	0.000945	0.595924
166	7555	CNBP	0.000618	0.593311
167	1826	DSCAM	0.001434	0.590168
168	4897	NRCAM	8.57E-08	0.5898
169	6716	SRD5A2	0.023964	0.587584
170	5251	PHEX	0.035669	0.586269
171	129642	MBOAT2	7.89E-07	0.585995
172	3742	GALNT8	7.60E-05	0.585665
173	2887	GRB10	6.85E-05	-0.58764
174	3712	IVD	2.29E-09	-0.58911
175	678	ZFP36L2	4.57E-07	-0.58959
176	1040	CDS1	0.010997	-0.59163
177	90141	EFCAB11	0.001098	-0.59238
178	1059	CENPB	0.000124	-0.5929
179	3090	HIC1	0.02802	-0.59372
180	29903	CCDC106	0.000434	-0.597
181	54361	WNT4	2.32E-05	-0.59749
182	81550	TDRD3	2.77E-06	-0.59837
183	8898	MTMR2	0.034402	-0.59882
184	1960	EGR3	0.000616	-0.6024
185	3339	HSPG2	0.001039	-0.60691
186	2155	F7	0.028365	-0.6085
187	11100	HNRNPUL1	0.017951	-0.60932
188	7123	CLEC3B	5.73E-08	-0.61301
189	9844	ELMO1	2.74E-05	-0.62222
190	5989	HGNC:9982	0.002238	-0.62388
191	6004	RGS16	9.76E-07	-0.6241
192	55957	LIN37	9.60E-05	-0.62709
193	5058	PAK1	0.008696	-0.62735
194	3298	HSF2	0.006881	-0.62939
195	50814	NSDHL	0.000144	-0.63025
196	51668	HSPB11	3.48E-05	-0.6305
197	6649	SOD3	0.036883	-0.63255
198	27445	PCLO	0.011926	-0.63624
199	8676	STX11	0.000467	-0.63697
200	5277	PIGA	0.006811	-0.64035

201	2619	GAS1	0.002816	-0.64075
202	4524	MTHFR	0.041206	-0.64918
203	27336	HTATSF1	0.00448	-0.64981
204	4493	MT1E	2.68E-05	-0.6512
205	7755	ZNF205	0.001924	-0.66042
206	3815	KIT	0.042131	-0.66656
207	2905	GRIN2C	5.96E-05	-0.66707
208	2009	EML1	4.00E-09	-0.66977
209	2788	GNG7	1.92E-06	-0.67215
210	10279	PRSS16	1.11E-05	-0.67225
211	23092	ARHGAP26	0.005073	-0.67536
212	28	ABO	0.016491	-0.67557
213	22849	CPEB3	2.33E-07	-0.67778
214	6480	ST6GAL1	0.000953	-0.67898
215	4007	PRICKLE3	0.03613	-0.67985
216	7849	PAX8	6.88E-05	-0.68524
217	25840	METTL7A	0.034275	-0.68544
218	10425	ARIH2	0.000363	-0.693
219	10100	TSPAN2	0.000702	-0.69332
220	5172	SLC26A4	3.62E-11	-0.69716
221	2273	FHL1	3.36E-09	-0.69808
222	670	BPHL	6.03E-05	-0.70171
223	84444	DOT1L	0.037573	-0.70555
224	4057	LTF	3.57E-10	-0.71261
225	11193	WBP4	0.001383	-0.71508
226	3081	HGD	9.27E-08	-0.72673
227	1734	DIO2	1.32E-10	-0.72726
228	5909	RAP1GAP	1.86E-09	-0.72971
229	26471	NUPR1	0.000304	-0.73394
230	255057	C19orf26	0.028982	-0.73829
231	6668	SP2	0.000589	-0.74243
232	6392	SDHD	0.001826	-0.7456
233	6844	VAMP2	0.018031	-0.74826
234	3213	HOXB3	0.016098	-0.75247
235	430	ASCL2	0.014075	-0.75314
236	5546	PRCC	1.43E-06	-0.76112
237	8857	FCGBP	1.18E-05	-0.76491
238	5897	RAG2	3.87E-05	-0.76635
239	1147	CHUK	0.000761	-0.76874
240	27350	APOBEC3C	8.26E-05	-0.77319
241	396	ARHGDIA	0.013364	-0.77533

242	7542	ZFPL1	0.001336	-0.77841
243	26146	TRAF3IP1	0.000189	-0.78888
244	762	CA4	3.05E-10	-0.79406
245	23443	SLC35A3	0.000171	-0.7947
246	25802	LMOD1	3.89E-08	-0.80358
247	6305	SBF1	1.13E-06	-0.80539
248	51232	CRIM1	7.84E-05	-0.81628
249	26112	CCDC69	0.015341	-0.82799
250	3399	ID3	2.91E-11	-0.83671
251	22927	HABP4	0.001917	-0.83713
252	2324	FLT4	4.54E-05	-0.83995
253	2618	GART	0.049823	-0.84525
254	2355	FOSL2	0.038951	-0.85493
255	4703	NEB	3.90E-06	-0.8582
256	432	ASGR1	2.01E-05	-0.89599
257	1805	DPT	0.00018	-0.8994
258	4494	MT1F	4.58E-09	-0.91087
259	219333	USP12	0.047108	-0.9167
260	2117	ETV3	0.000167	-0.93059
261	6722	SRF	0.003049	-0.94275
262	1381	CRABP1	1.48E-06	-0.95542
263	6921	TCEB1	0.004592	-0.98698
264	2323	FLT3LG	0.009582	-0.98782
265	1299	COL9A3	8.03E-05	-1.00485
266	4713	NDUFB7	0.000215	-1.00738
267	4495	MT1G	1.39E-07	-1.05177
268	9265	CYTH3	7.71E-05	-1.07064
269	8458	TTF2	0.030282	-1.09564
270	968	CD68	0.007163	-1.11098
271	6624	FSCN1	0.003741	-1.12761
272	4920	ROR2	3.74E-05	-1.19808
273	2167	FABP4	8.24E-10	-1.24181
274	744	MPPED2	1.02E-13	-1.25312
275	3292	HSD17B1	1.63E-05	-1.28357
276	1014	CDH16	3.65E-16	-1.33575
277	1733	DIO1	8.64E-07	-1.42927
278	7173	TPO	3.90E-15	-1.49917
279	9351	SLC9A3R2	0.00174	-1.61953

Supplementary Table S2. The Gene Ontology (GO) enrichment analysis was performed to identify the key biological annotations for malignant and benign thyroid nodules, including BP (biological process), MF (molecular function) and CC (cell component).

GO ID	Description	Raw *p*-value	Gene Name	No.
			**BP (Biological Process)**	
GO:0042445	hormone metabolic process	1.54E-06	SCG5/TSHB/TTR/CPA3/TIPARP/ALDH1A3/CYP1B1/SRD5A2/WNT4/PAX8/DIO2/CRABP1/HSD17B1/DIO1/TPO	15
GO:0002576	platelet degranulation	1.66E-05	PROS1/FN1/APOH/CD63/TIMP1/CD9/SERPINA1/PF4/CLEC3B/HABP4	10
GO:0035924	cellular response to vascular endothelial growth factor stimulus	5.46E-05	ANXA1/XDH/CD63/EGR3/GAS1/FLT4/MT1G	7
GO:0048066	developmental pigmentation	5.53E-05	CITED1/RAB32/RAB27A/CD63/TYR/KIT	6
GO:0048608	reproductive structure development	5.74E-05	CITED1/ANXA1/ALKBH1/NRIP1/TIPARP/BASP1/KRT19/PLAG1/WNT2/AMHR2/SRD5A2/WNT4/NSDHL/KIT/NUPR1/ASCL2/COL9A3/ROR2	18
GO:0061458	reproductive system development	6.28E-05	CITED1/ANXA1/ALKBH1/NRIP1/TIPARP/BASP1/KRT19/PLAG1/WNT2/AMHR2/SRD5A2/WNT4/NSDHL/KIT/NUPR1/ASCL2/COL9A3/ROR2	18
GO:0006590	thyroid hormone generation	8.65E-05	PAX8/DIO2/DIO1/TPO	4
GO:0060231	mesenchymal to epithelial transition	1.38E-04	CITED1/BASP1/WNT4/PAX8	4
GO:0050931	pigment cell differentiation	1.56E-04	CITED1/RAB32/RAB27A/CD63/KIT	5
GO:0042403	thyroid hormone metabolic process	2.08E-04	PAX8/DIO2/DIO1/TPO	4
GO:0043312	neutrophil degranulation	2.50E-04	QPCT/RAP1A/RAB27A/CD63/TTR/SERPINA1/CD44/TCN1/VAPA/NPC2/SERPINB10/COPB1/EPX/ARPC5/BPI/METTL7A/LTF/CD68	18
GO:0002283	neutrophil activation involved in immune response	2.70E-04	QPCT/RAP1A/RAB27A/CD63/TTR/SERPINA1/CD44/TCN1/VAPA/NPC2/SERPINB10/COPB1/EPX/ARPC5/BPI/METTL7A/LTF/CD68	18

GO:0010951	negative regulation of endopeptidase activity	2.88E-04	PROS1/TIMP1/SERPINA1/CD44/PZP/SPOCK1/SERPINB10/SERPINA7/ITIH2/GAPDH/LTF/CRIM1	12
GO:0042119	neutrophil activation	3.44E-04	QPCT/RAP1A/RAB27A/CD63/TTR/SERPINA1/CD44/TCN1/VAPA/NPC2/SERPINB10/COPB1/EPX/ARPC5/BPI/METTL7A/LTF/CD68	18
GO:0002446	neutrophil mediated immunity	3.52E-04	QPCT/RAP1A/RAB27A/CD63/TTR/SERPINA1/CD44/TCN1/VAPA/NPC2/SERPINB10/COPB1/EPX/ARPC5/BPI/METTL7A/LTF/CD68	18
GO:0010466	negative regulation of peptidase activity	4.39E-04	PROS1/TIMP1/SERPINA1/CD44/PZP/SPOCK1/SERPINB10/SERPINA7/ITIH2/GAPDH/LTF/CRIM1	12
GO:0031346	positive regulation of cell projection organization	4.69E-04	TENM1/RAP1A/FN1/MAPK6/EPS8/DPYSL3/CNTN1/CAPRIN1/DSCAM/PAK1/KIT/CPEB3/ARHGDIA/SRF/FSCN1	15
GO:0008585	female gonad development	4.77E-04	NRIP1/TIPARP/AMHR2/WNT4/KIT/NUPR1/COL9A3	7
GO:0032970	regulation of actin filament-based process	5.36E-04	TENM1/CIT/EPS8/TMSB10/KCNJ2/DSG2/SDC4/S100A10/ARPC5/WNT4/PAK1/ARHGDIA/LMOD1/NEB/FSCN1	15
GO:0043473	pigmentation	5.40E-04	CITED1/CDH3/RAB32/RAB27A/CD63/TYR/KIT	7
GO:0034754	cellular hormone metabolic process	5.68E-04	TTR/TIPARP/ALDH1A3/CYP1B1/SRD5A2/WNT4/CRABP1/HSD17B1	8
GO:0035019	somatic stem cell population maintenance	5.97E-04	NR2E1/POLR2B/ZFP36L2/KIT/PAX8/ASCL2	6
GO:0046545	development of primary female sexual characteristics	6.48E-04	NRIP1/TIPARP/AMHR2/WNT4/KIT/NUPR1/COL9A3	7
GO:0018958	phenol-containing compound metabolic process	7.28E-04	CITED1/CDH3/TYR/PAX8/DIO2/DIO1/TPO	7
GO:0051491	positive regulation of filopodium assembly	8.60E-04	TENM1/DPYSL3/SRF/FSCN1	4
GO:0034329	cell junction assembly	9.18E-04	PTPRO/CDH3/RAP1A/FN1/CD9/SDC4/S100A10/GABRA1/AMIGO2/DSCAM/NRCAM/WNT4/PCLO/SRF/FSCN1	15
GO:0061005	cell differentiation involved in kidney development	1.06E-03	PTPRO/CITED1/BASP1/WNT4/PAX8	5

GO:0003338	metanephros morphogenesis	1.10E-03	CITED1/BASP1/WNT4/PAX8	4
GO:0010273	detoxification of copper ion	1.18E-03	MT1E/MT1F/MT1G	3
GO:0090136	epithelial cell-cell adhesion	1.18E-03	CYP1B1/KIT/SRF	3
GO:1990169	stress response to copper ion	1.18E-03	MT1E/MT1F/MT1G	3
GO:0030282	bone mineralization	1.32E-03	COL1A2/NBR1/PHEX/WNT4/CLEC3B/LTF/ROR2	7
GO:0046660	female sex differentiation	1.39E-03	NRIP1/TIPARP/AMHR2/WNT4/KIT/NUPR1/COL9A3	7
GO:0048568	embryonic organ development	1.44E-03	CITED1/KRT19/ALDH1A3/WNT2/NSDHL/KIT/PAX8/HOXB3/ASCL2/TRAF3IP1/ID3/SRF/FLT3LG/ROR2/TPO	15
GO:0032956	regulation of actin cytoskeleton organization	1.49E-03	TENM1/CIT/EPS8/TMSB10/SDC4/S100A10/ARPC5/WNT4/PAK1/ARHGDIA/LMOD1/NEB/FSCN1	13
GO:0042446	hormone biosynthetic process	1.52E-03	SRD5A2/WNT4/DIO2/HSD17B1/DIO1/TPO	6
GO:0051017	actin filament bundle assembly	1.72E-03	EPS8/SDC4/S100A10/DPYSL3/WNT4/PAK1/SRF/FSCN1	8
GO:0061687	detoxification of inorganic compound	1.73E-03	MT1E/MT1F/MT1G	3
GO:0097501	stress response to metal ion	1.73E-03	MT1E/MT1F/MT1G	3
GO:0071276	cellular response to cadmium ion	1.91E-03	MT1E/CHUK/MT1F/MT1G	4
GO:0048857	neural nucleus development	1.98E-03	ATP5PB/S100A1/BASP1/ALDH1A3/CNP	5
GO:0061572	actin filament bundle organization	2.02E-03	EPS8/SDC4/S100A10/DPYSL3/WNT4/PAK1/SRF/FSCN1	8
GO:0072077	renal vesicle morphogenesis	2.06E-03	CITED1/WNT4/PAX8	3
GO:0007015	actin filament organization	2.08E-03	TENM1/CIT/EPS8/TMSB10/SDC4/S100A10/DPYSL3/ARPC5/WNT4/PAK1/LMOD1/NEB/SRF/FSCN1	14

GO:0048009	insulin-like growth factor receptor signaling pathway	2.12E-03	CDH3/PHIP/IGFBP6/CRIM1	4
GO:0046686	response to cadmium ion	2.13E-03	MT1E/KIT/CHUK/MT1F/MT1G	5
GO:0045861	negative regulation of proteolysis	2.17E-03	PROS1/TIMP1/SERPINA1/CD44/PZP/SPOCK1/SERPINB10/SERPINA7/ITIH2/GAPDH/GAS1/LTF/CRIM1	13
GO:0045785	positive regulation of cell adhesion	2.23E-03	ANXA1/DPP4/FN1/AGR2/TFRC/CD44/ADAM9/SDC4/S100A10/CD40LG/WNT4/EGR3/PAK1/CYTH3	14
GO:0072087	renal vesicle development	2.42E-03	CITED1/WNT4/PAX8	3
GO:0031032	actomyosin structure organization	2.45E-03	CIT/SDC4/S100A10/KRT19/WNT4/PAK1/LMOD1/NEB/SRF	9
		**MF (Molecular Function)**		
GO:0004866	endopeptidase inhibitor activity	9.45E-05	PROS1/TIMP1/SERPINA1/PZP/SPOCK1/SERPINB10/SERPINA7/ITIH2/GAPDH/LTF/CRIM1	11
GO:0030414	peptidase inhibitor activity	1.34E-04	PROS1/TIMP1/SERPINA1/PZP/SPOCK1/SERPINB10/SERPINA7/ITIH2/GAPDH/LTF/CRIM1	11
GO:0061135	endopeptidase regulator activity	1.34E-04	PROS1/TIMP1/SERPINA1/PZP/SPOCK1/SERPINB10/SERPINA7/ITIH2/GAPDH/LTF/CRIM1	11
GO:0061134	peptidase regulator activity	1.69E-04	PROS1/FN1/TIMP1/SERPINA1/PZP/SPOCK1/SERPINB10/SERPINA7/ITIH2/GAPDH/LTF/CRIM1	12
GO:0005539	glycosaminoglycan binding	2.55E-04	FSTL1/TENM1/FN1/NELL2/ZNF207/APOH/CD44/PF4/DPYSL3/CLEC3B/SOD3/LTF	12
GO:0008201	heparin binding	3.19E-04	FSTL1/TENM1/FN1/NELL2/ZNF207/APOH/PF4/CLEC3B/SOD3/LTF	10
GO:1901681	sulfur compound binding	5.63E-04	FSTL1/TENM1/DBI/FN1/NELL2/ZNF207/APOH/PF4/DPYSL3/CLEC3B/SOD3/LTF	12
GO:0004867	serine-type endopeptidase inhibitor activity	6.56E-04	SERPINA1/PZP/SPOCK1/SERPINB10/SERPINA7/ITIH2/CRIM1	7

GO:0005001	transmembrane receptor protein tyrosine phosphatase activity	2.15E-03	PTPRO/PTPRT/PTPRE	3
GO:0019198	transmembrane receptor protein phosphatase activity	2.15E-03	PTPRO/PTPRT/PTPRE	3
GO:0005518	collagen binding	3.85E-03	FN1/CD44/SPOCK1/ADAM9/PAK1	5
GO:0002020	protease binding	3.89E-03	DPP4/FN1/TIMP1/COL1A2/SERPINA1/PZP/KIT	7
GO:0008017	microtubule binding	5.27E-03	ZNF207/POLB/KIF1B/VAPA/CETN1/CKAP5/GAPDH/EML1/TRAF3IP1/CCDC69	10
GO:0004857	enzyme inhibitor activity	6.02E-03	ANXA1/PROS1/SCG5/TIMP1/SERPINA1/PZP/SPOCK1/SERPINB10/SERPINA7/ITIH2/GAPDH/LTF/CRIM1	13
GO:0015631	tubulin binding	6.52E-03	ZNF207/POLB/OFD1/KIF1B/VAPA/CETN1/CCT5/CKAP5/GAPDH/EML1/TRAF3IP1/CCDC69	12
GO:0035925	mRNA 3'-UTR AU-rich region binding	7.41E-03	ELAVL1/ZFP36L2/CPEB3	3
GO:0098632	cell-cell adhesion mediator activity	7.50E-03	ANXA1/DSG2/DSCAM/NRCAM	4
GO:0019199	transmembrane receptor protein kinase activity	7.73E-03	AMHR2/KIT/CRIM1/FLT4/ROR2	5
GO:0017091	AU-rich element binding	9.13E-03	ELAVL1/ZFP36L2/CPEB3	3
GO:0033764	steroid dehydrogenase activity, acting on the CH-OH group of donors, NAD or NADP as acceptor	1.01E-02	SRD5A2/NSDHL/HSD17B1	3
GO:0050839	cell adhesion molecule binding	1.16E-02	PTPRO/ANXA1/CDH3/TENM1/FN1/TXNDC9/TJP2/CD9/VAPA/DSG2/ADAM9/PTPRT/CKAP5/FSCN1/SLC9A3R2	15
GO:0098631	cell adhesion mediator activity	1.33E-02	ANXA1/DSG2/DSCAM/NRCAM	4
GO:0004126	cytidine deaminase activity	1.43E-02	APOBEC2/APOBEC3C	2

GO:0008239	dipeptidyl-peptidase activity	1.43E-02	DPP4/PRSS16	2
GO:0004714	transmembrane receptor protein tyrosine kinase activity	1.57E-02	KIT/CRIM1/FLT4/ROR2	4
GO:0003906	DNA-(apurinic or apyrimidinic site) endonuclease activity	1.68E-02	POLB/ALKBH1	2
GO:0052744	phosphatidylinositol monophosphate phosphatase activity	1.68E-02	INPP5F/MTMR2	2
GO:0086080	protein binding involved in heterotypic cell-cell adhesion	1.68E-02	DSG2/NRCAM	2
GO:0016229	steroid dehydrogenase activity	1.68E-02	SRD5A2/NSDHL/HSD17B1	3
GO:0004725	protein tyrosine phosphatase activity	1.99E-02	PTPRO/DUSP6/PTPRT/PTPRE/MTMR2	5
GO:0019838	growth factor binding	2.02E-02	COL1A2/IGFBP6/AMHR2/RHBDF1/CRIM1/FLT4	6
GO:0004089	carbonate dehydratase activity	2.21E-02	CA6/CA4	2
GO:0005109	frizzled binding	2.25E-02	WNT2/WNT4/ROR2	3
GO:0008236	serine-type peptidase activity	2.39E-02	DPP4/TMPRSS15/CFI/TMPRSS11D/F7/PRSS16/LTF	7
GO:0008191	metalloendopeptidase inhibitor activity	2.50E-02	TIMP1/SPOCK1	2
GO:0016825	hydrolase activity, acting on acid phosphorus-nitrogen bonds	2.65E-02	DPP4/TMPRSS15/CFI/TMPRSS11D/F7/PRSS16/LTF	7
GO:0017171	serine hydrolase activity	2.65E-02	DPP4/TMPRSS15/CFI/TMPRSS11D/F7/PRSS16/LTF	7
GO:0015125	bile acid transmembrane transporter activity	2.81E-02	ABCC3/SLC10A1	2
GO:0042826	histone deacetylase binding	2.96E-02	NR2E1/NRIP1/HIC1/SP2/SRF	5

GO:0038024	cargo receptor activity	3.06E-02	TMPRSS15/TFRC/CFI/ASGR1	4
GO:0008138	protein tyrosine/serine/threonine phosphatase activity	3.26E-02	DUSP6/MTMR2/SBF1	3
GO:0005086	ARF guanyl-nucleotide exchange factor activity	3.46E-02	PSD3/CYTH3	2
GO:0016500	protein-hormone receptor activity	3.46E-02	AMHR2/PAX8	2
GO:0031681	G-protein beta-subunit binding	3.46E-02	CCT5/GNG7	2
GO:0045296	cadherin binding	3.47E-02	PTPRO/ANXA1/CDH3/TXNDC9/TJP2/VAPA/PTPRT/CKAP5/FSCN1/SLC9A3R2	10
GO:0008373	sialyltransferase activity	3.80E-02	ST3GAL5/ST6GAL1	2
GO:0030506	ankyrin binding	3.80E-02	RHAG/NRCAM	2
GO:0004252	serine-type endopeptidase activity	3.91E-02	DPP4/TMPRSS15/CFI/TMPRSS11D/F7/LTF	6
GO:0030674	protein-macromolecule adaptor activity	4.33E-02	ANXA1/TRIM22/EPS8/TJP2/COL1A2/STX11/FSCN1	7
GO:0005158	insulin receptor binding	4.53E-02	PHIP/GRB10	2
			**CC (Cell Component)**	
GO:0062023	collagen-containing extracellular matrix	4.69E-06	ANXA1/MXRA7/FN1/APOH/TIMP1/COL1A2/CPA3/SERPINA1/PZP/PF4/S100A10/WNT2/ITIH2/HSPG2/F7/CLEC3B/SOD3/DPT/COL9A3	19
GO:0034774	secretory granule lumen	1.23E-05	PROS1/QPCT/FN1/APOH/RAB27A/TIMP1/TTR/SERPINA1/PF4/TCN1/NPC2/EPX/ARPC5/BPI/CLEC3B/LTF	16
GO:0060205	cytoplasmic vesicle lumen	1.44E-05	PROS1/QPCT/FN1/APOH/RAB27A/TIMP1/TTR/SERPINA1/PF4/TCN1/NPC2/EPX/ARPC5/BPI/CLEC3B/LTF	16
GO:0031983	vesicle lumen	1.55E-05	PROS1/QPCT/FN1/APOH/RAB27A/TIMP1/TTR/SERPINA1/PF4/TCN1/NPC2/EPX/ARPC5/BPI/CLEC3B/LTF	16

GO:0016324	apical plasma membrane	5.57E-04	PTPRO/ANXA1/DPP4/FN1/RAB27A/CD9/CD44/SLC17A4/DSG2/PRKG2/SLC26A4/CA4/SLC9A3R2	13
GO:0033162	melanosome membrane	8.72E-04	RAB32/RAB27A/TYR	3
GO:0045009	chitosome	8.72E-04	RAB32/RAB27A/TYR	3
GO:0090741	pigment granule membrane	8.72E-04	RAB32/RAB27A/TYR	3
GO:0071437	invadopodium	1.31E-03	DPP4/PAK1/FSCN1	3
GO:0035580	specific granule lumen	1.72E-03	QPCT/RAB27A/TCN1/BPI/LTF	5
GO:0031091	platelet alpha granule	1.73E-03	PROS1/FN1/TIMP1/CD9/SERPINA1/PF4	6
GO:0031093	platelet alpha granule lumen	2.43E-03	PROS1/FN1/TIMP1/SERPINA1/PF4	5
GO:0030426	growth cone	2.81E-03	PTPRO/EPS8/BASP1/DPYSL3/ARPC5/DSCAM/CBARP/FSCN1	8
GO:0042827	platelet dense granule	2.96E-03	APOH/CD63/CLEC3B	3
GO:0045177	apical part of cell	3.01E-03	PTPRO/ANXA1/DPP4/FN1/RAB27A/CD9/CD44/SLC17A4/DSG2/PRKG2/SLC26A4/CA4/SLC9A3R2	13
GO:0005796	Golgi lumen	3.07E-03	PROS1/SDC4/WNT4/HSPG2/F7/SOD3	6
GO:0030667	secretory granule membrane	3.23E-03	RAP1A/RAB27A/CD63/CD9/CD44/VAPA/SERPINB10/COPB1/VAMP2/CA4/CD68	11
GO:0030427	site of polarized growth	3.36E-03	PTPRO/EPS8/BASP1/DPYSL3/ARPC5/DSCAM/CBARP/FSCN1	8
GO:0009897	external side of plasma membrane	3.66E-03	ANXA1/KLRD1/TFRC/CD9/CD1B/ADAM9/ALCAM/CD40LG/WNT2/NRCAM/KIT/CA4/FLT3LG	13
GO:0042470	melanosome	3.72E-03	RAB32/TFRC/RAB27A/CD63/TYR/CNP	6
GO:0048770	pigment granule	3.72E-03	RAB32/TFRC/RAB27A/CD63/TYR/CNP	6
GO:0005788	endoplasmic reticulum lumen	4.24E-03	FSTL1/DBI/FN1/TIMP1/COL1A2/SERPINA1/APOL1/ITIH2/WNT4/F7/COL9A3	11

GO:0031233	intrinsic component of external side of plasma membrane	4.37E-03	ADAM9/CA4/FLT3LG	3
GO:0072562	blood microparticle	4.62E-03	C4BPA/PROS1/FN1/TFRC/PZP/APOL1/ITIH2	7
GO:0005925	focal adhesion	4.70E-03	ANXA1/DPP4/CD9/CD44/ADAM9/SDC4/ALCAM/ARPC5/HSPG2/PAK1/ARHGAP26/FHL1/SLC9A3R2	13
GO:0030055	cell-substrate junction	5.41E-03	ANXA1/DPP4/CD9/CD44/ADAM9/SDC4/ALCAM/ARPC5/HSPG2/PAK1/ARHGAP26/FHL1/SLC9A3R2	13
GO:1904724	tertiary granule lumen	7.23E-03	QPCT/TCN1/METTL7A/LTF	4
GO:0017146	NMDA selective glutamate receptor complex	9.81E-03	EPS8/GRIN2C	2
GO:0032585	multivesicular body membrane	1.17E-02	RAB27A/CD63	2
GO:0010494	cytoplasmic stress granule	1.36E-02	ELAVL1/DYRK3/CAPRIN1/HABP4	4
GO:0030016	myofibril	1.36E-02	S100A1/SDC4/KRT19/NBR1/PAK1/LMOD1/HABP4/NEB	8
GO:0032045	guanyl-nucleotide exchange factor complex	1.37E-02	RAP1A/ELMO1	2
GO:0031089	platelet dense granule lumen	1.58E-02	APOH/CLEC3B	2
GO:0030136	clathrin-coated vesicle	1.66E-02	TFRC/RAB27A/CD9/INPP5F/TGFA/VAMP2/ROR2	7
GO:0043292	contractile fiber	1.72E-02	S100A1/SDC4/KRT19/NBR1/PAK1/LMOD1/HABP4/NEB	8
GO:0030027	lamellipodium	1.89E-02	PTPRO/DPP4/CD44/DPYSL3/ARPC5/PAK1/FSCN1	7
GO:0150034	distal axon	1.90E-02	PTPRO/EPS8/BASP1/DPYSL3/ARPC5/DSCAM/KCNA6/CBARP/FSCN1	9
GO:0030135	coated vesicle	2.06E-02	TFRC/RAB27A/CD9/SERPINA1/COPB1/INPP5F/TGFA/VAMP2/ROR2	9
GO:0005766	primary lysosome	2.17E-02	CD63/TTR/VAPA/NPC2/BPI/CD68	6

GO:0042582	azurophil granule	2.17E-02	CD63/TTR/VAPA/NPC2/BPI/CD68	6
GO:0030133	transport vesicle	2.22E-02	RAB27A/CPA3/OVGP1/DPYSL3/COPB1/TGFA/MTMR2/STX11/CBARP/VAMP2/CA4	11
GO:0030665	clathrin-coated vesicle membrane	2.26E-02	TFRC/CD9/TGFA/VAMP2/ROR2	5
GO:0016607	nuclear speck	2.41E-02	TENM1/TRIM22/NRIP1/BASP1/DYRK3/MBD1/PLAG1/PNN/WBP4/PRCC/HABP4	11
GO:0030017	sarcomere	2.47E-02	S100A1/KRT19/NBR1/PAK1/LMOD1/HABP4/NEB	7
GO:0099572	postsynaptic specialization	2.47E-02	PTPRO/EPS8/PSD3/SPOCK1/GABRA1/NRCAM/MTMR2/PCLO/GRIN2C/CPEB3	10
GO:0042581	specific granule	2.49E-02	QPCT/RAP1A/RAB27A/TCN1/BPI/LTF	6
GO:0016528	sarcoplasm	2.56E-02	XDH/S100A1/SPOCK1/HABP4	4
GO:0031252	cell leading edge	2.65E-02	PTPRO/DPP4/EPS8/PSD3/CD44/DPYSL3/ARPC5/GABRA1/PAK1/CYTH3/FSCN1	11
GO:0005811	lipid droplet	2.67E-02	GAPDH/NSDHL/METTL7A/FABP4	4
GO:0070820	tertiary granule	2.77E-02	QPCT/TCN1/SERPINB10/COPB1/METTL7A/LTF	6

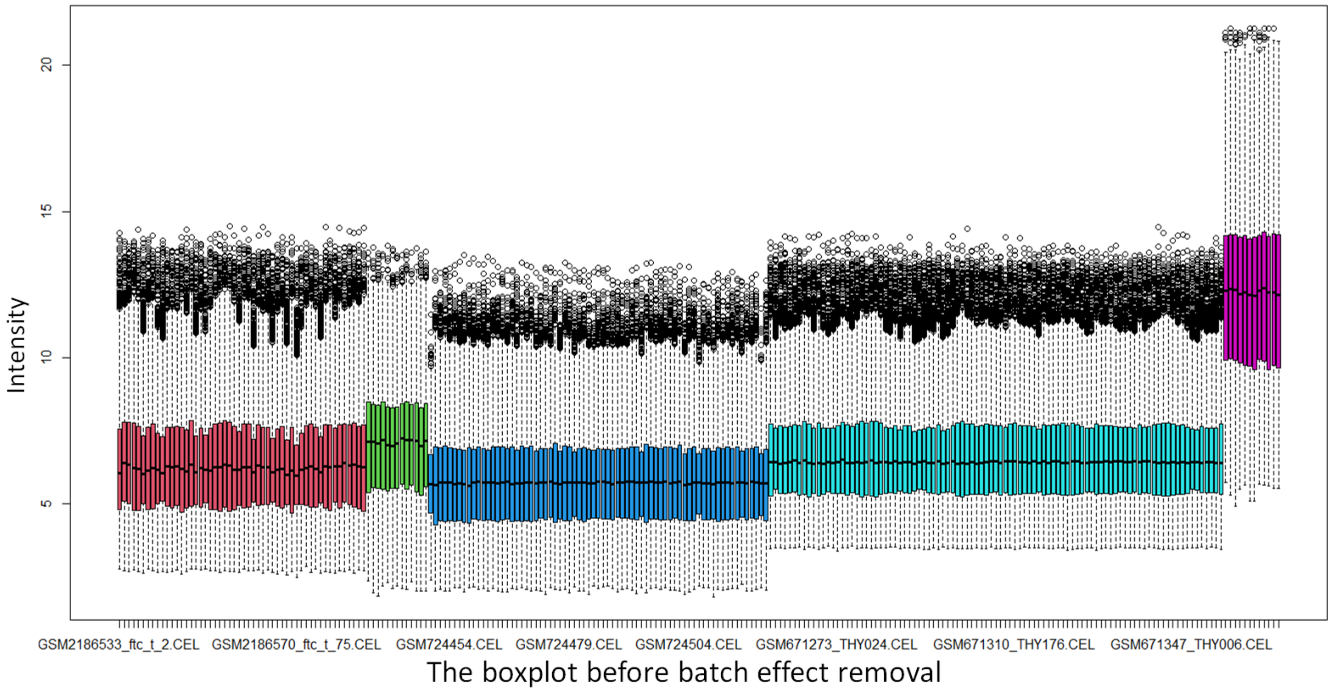

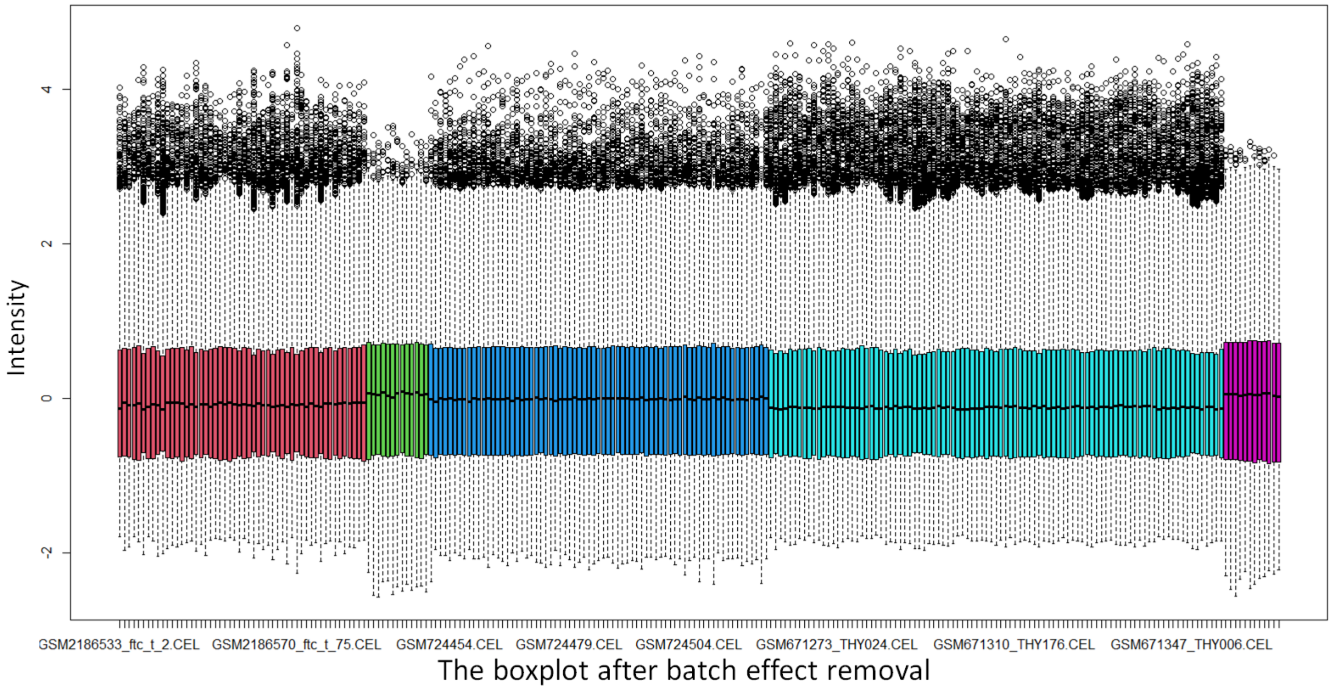

Supplementary Figure S1. The boxplots of the samples before and after batch effect removal for the five transcriptomic datasets (as shown in **Table 1**).
